# Risk factors associated with the failure of day surgery for total knee arthroplasty a multivariate logistic regression analysis

**DOI:** 10.3389/fsurg.2025.1597068

**Published:** 2025-06-26

**Authors:** Pan Luo, Jiabin Feng, Yankun Li, Yingli Zou, Xuepeng Zhu, Li Sun, Bo Li

**Affiliations:** Department of Orthopaedic Surgery, Guizhou Provincial People’s Hospital, Guiyang, Guizhou, China

**Keywords:** day surgery, delayed discharge, joint arthroplasty, knee joint, risk factors

## Abstract

**Objective:**

To explore and analyze the risk factors leading to delayed discharge in patients undergoing day surgery for total knee arthroplasty, providing a theoretical basis for optimizing patient selection, targeted interventions, and managing expectations and risks of day surgery patients.

**Methods:**

A retrospective analysis of clinical data from patients diagnosed with “knee osteoarthritis” and scheduled for day surgery for total knee arthroplasty at the Department of Orthopedics, Guizhou Provincial People's Hospital from October 2021 to June 2023. Patients were divided into a normal discharge group and a delayed discharge group based on whether their hospital stay exceeded 48 h. Univariate analysis and multivariate logistic regression analysis were used to identify potential risk factors between the two groups.

**Results:**

Among the 233 patients scheduled for day surgery for total knee arthroplasty, 82 experienced delayed discharge. Univariate analysis showed that age (*P* = 0.042), glomerular filtration rate (*P* = 0.011), diabetes history (*P* = 0.046), disease duration (*P* = 0.012), knee flexion range of motion (*P* < 0.001), knee flexion contracture degree (*P* = 0.006), and surgery time (*P* = 0.002) had statistically significant differences between the two groups. Multivariate logistic regression analysis revealed that disease duration (*P* = 0.017), knee flexion range of motion (*P* < 0.001), glomerular filtration rate (*P* = 0.010), and surgery time (*P* = 0.020) were independent risk factors. Additionally, postoperative motor function decline or sensory impairment, pain, dizziness or orthostatic hypotension, postoperative limb swelling, postoperative nausea and vomiting, bleeding and exudation, and psychosocial factors were the main reasons for delayed discharge.

**Conclusions:**

Our study found that disease duration, knee flexion range of motion, glomerular filtration rate, and surgery time are independent risk factors for delayed discharge after day surgery for total knee arthroplasty. Patients with a disease duration of more than 8 years, preoperative flexion range of motion less than 90°, and low estimated glomerular filtration rate may not be suitable for day surgery for total knee arthroplasty. Surgeons should make adequate preoperative preparations and plans, refine surgical skills, and shorten surgery time under the premise of ensuring surgical effects to reduce the risk of postoperative delayed discharge.

## 1 Introduction

In China, knee osteoarthritis (OA) is a highly prevalent degenerative joint disease, with an increasing incidence rate due to the acceleration of population aging. The aetiopathogenesis of KOA involves both primary and secondary mechanisms. Primary OA is characterized by progressive synovitis and joint tissue degradation, where synovial-derived inflammatory mediators such as IL-6, IL-8, and MMPs play a central role in driving cartilage destruction and disease progression (1). Secondary OA often arises from traumatic joint injuries, with post-traumatic osteoarthritis (PTOA) developing in up to 50% of patients following articular fractures due to persistent articular step-off and malalignment despite surgical intervention (2). Due to the pain and functional impairment caused by the disease, knee OA has become one of the main causes of disability in the middle-aged and elderly. Many patients eventually need to choose surgical treatment to alleviate pain and restore joint function when non-surgical treatments (such as drug therapy, weight management, and physical therapy) fail to effectively relieve symptoms.

However, surgical treatment often means expensive medical costs and a long recovery process. Total knee arthroplasty (TKA), as an effective surgical method for treating late-stage knee OA, requires patients to stay in the hospital for a long time for observation and rehabilitation training after surgery.

Although TKA surgery can significantly improve the function of the knee joint, postoperative complication risk management is a key challenge to improving the surgical outcome. Patients may face risks such as infection, thrombosis, joint stiffness, and prosthesis loosening (3, 4). Therefore, strict postoperative monitoring and regular follow-ups are crucial for ensuring patient safety and promoting postoperative recovery. In addition, with in-depth research, the development of minimally invasive surgical methods and new prosthesis materials is continuously improving the success rate of TKA, reducing surgical trauma, and shortening recovery time (5–7).

Day surgery, also known as outpatient surgery, generally refers to a medical service model where patients are admitted, undergo surgery, and are discharged on the same day (8, 9). In 2003, the International Day Surgery Association defined day surgery as a process within a working day (not exceeding 24 h of hospital stay) where patients can complete admission, surgery, and discharge in sequence, excluding outpatient surgeries in hospitals or clinics. By 2015, the China Day Surgery Cooperation Alliance had localized the definition of day surgery: patients are arranged to complete preoperative examinations and anesthesia assessments before admission, and surgery time appointments, and can be admitted and undergo surgery on the same day. For cases that require extended hospital stays due to medical conditions, the maximum hospital stay shall not exceed 48 h (9, 10).

So far, there is still a divergence in the international definition of day surgery for total knee arthroplasty. Some countries or regions define day surgery as patients discharged on the same day (11, 12), or patients who stay in the hospital for one night (13), or patients who stay in the hospital for at most two nights (14, 15), or simply state their status without a clear definition (16, 17). In the United States, the reimbursement ratio for inpatient and day surgeries differs greatly, and the definition of day surgery mainly depends on the medical insurance classification of the patient's surgery by the doctor. Inappropriate classification of non-day surgery patients can lead to huge economic losses. Many private insurance companies have extended the standard of day surgery from 23 h to 48 h (18). In China, the understanding and implementation of day surgery tend to extend the patient's recovery period treatment model (19). The definition of day surgery for total knee arthroplasty within 48 h of admission and discharge is in line with China's medical environment.

Day surgery for total knee arthroplasty has been proven to be very successful and safe (20–22). This mode of total knee arthroplasty can significantly improve patient satisfaction, facilitate rapid recovery, reduce medical costs, increase bed turnover rate, and save medical insurance costs.

Multiple studies (23–25) have reported that the success rate of same-day discharge from outpatient surgery centers or large-volume hospitals is as high as 80%–100%. However, these studies usually involve strictly selected patients and exclude those with comorbidities, advanced age, or high body mass index. Although day surgery for total knee arthroplasty is obviously achievable, the results of these studies are unlikely to be generalized to all hospitals. In a prospective two-center study, only about 15% of 557 patients who underwent joint replacement day surgery achieved discharge on the same day after surgery (26). This huge difference highlights the need to identify specific risk factors for delayed discharge in day surgery for total knee arthroplasty.

Although it may not be realistic for the most experienced joint replacement surgeons or medical institutions to ensure that all patients undergoing day surgery for total knee arthroplasty can be discharged on time, we have noticed that there may be some modifiable risk factors for postoperative timely discharge. Therefore, we conducted a retrospective analysis of the clinical data of patients scheduled for day surgery for total knee arthroplasty to explore and analyze the risk factors affecting postoperative delayed discharge. By identifying these risk factors, we can better optimize patient selection or carry out necessary interventions to improve the efficiency of timely discharge. At the same time, this helps to develop more complete prediction models or preoperative scoring standards in the future to manage the expectations and risks of day surgery patients.

## 2 Methods

### 2.1 Patient data

#### 2.1.1 Research subjects and grouping

Clinical data were collected from cases admitted to the Department of Orthopedics, Guizhou Provincial People's Hospital for day surgery for total knee arthroplasty from October 2021 to June 2023. The surgery was performed by the same chief surgeon. Patients were divided into two categories based on whether their hospital stay exceeded 48 h: the normal discharge group and the delayed discharge group.

#### 2.1.2 Inclusion criteria

1. Patients with complete medical records required for this study.
2. Patients scheduled for day surgery for total knee arthroplasty.

#### 2.1.3 Exclusion criteria

1. Patients with a primary diagnosis other than knee osteoarthritis.
2. Patients undergoing simultaneous bilateral total knee arthroplasty.

## 3 Day surgery for total knee arthroplasty process

### 3.1 Inclusion and exclusion criteria for day surgery for total knee arthroplasty patients

#### 3.1.1 Inclusion criteria

1. In principle, patients aged 65 years or younger; patients over 65 years old with conditions may be included after anesthesia assessment and departmental preoperative discussion.
2. Patients who meet the indications for primary artificial knee replacement and are willing to undergo day artificial joint replacement.
3. Patients with ASA anesthesia grading of I–II.
4. Patients have family members who can guide and supervise functional training after surgery and are adapted to the home rehabilitation environment.
5. No communication barriers, and patients or their caregivers can skillfully use mobile phones and other communication tools.
6. BMI ≤ 30 m^2^/kg; preoperative blood pressure ≤140/90 mmHg for three consecutive days; preoperative fasting blood glucose ≤8 mmol/L and postprandial blood glucose ≤11.1 mmol/L, glycated hemoglobin ≤8 mmol/L; albumin ≥35 g/L; Hb ≥100 g/L.

#### 3.1.2 Exclusion criteria

1. Patients with severe underlying diseases, such as cardiovascular and pulmonary diseases, chronic kidney disease.
2. Patients with a history of heart failure, myocardial infarction, severe pulmonary diseases, and cerebrovascular accidents.
3. Patients who cannot return home immediately after surgery, have poor self-care abilities, are overly dependent on others, or have psychological diseases.
4. Patients with poor preoperative cognitive abilities, hospital dependency mentality, fear of home rehabilitation, unstable mentality, and emotional fluctuations.
5. Patients with unstable preoperative blood glucose and blood pressure control.

### 3.2 Pre-admission process

Patients go through the pre-admission process, during which the costs incurred are included in the hospitalization process. Patients do not need to stay in the ward during this period and only need to complete relevant examinations and outpatient assessments. If patients have abnormal preoperative results or surgical contraindications and cannot undergo day surgery, they will exit the pre-admission process.

#### 3.2.1 Preoperative examinations and anesthesia assessment

Outpatient doctors screen patients according to the inclusion and exclusion criteria and assist in handling pre-admission procedures. The attending physician issues or completes relevant examination application forms.

Imaging examinations include: standing anteroposterior and lateral knee DR, patellar axial DR, full-length anteroposterior and lateral lower limb DR, routine 12-channel electrocardiogram, routine cardiac ultrasound examination, left ventricular systolic and diastolic function determination, pulmonary (mediastinal) CT plain scan three-dimensional imaging, transcranial Doppler, bilateral lower limb artery and vein color Doppler ultrasound, etc.

Laboratory examinations: complete blood count, liver function, kidney function, electrolytes, blood glucose, preoperative four infectious disease screenings, coagulation mechanism four items, dynamic monitoring of coagulation function and platelet function, erythrocyte sedimentation rate, C-reactive protein, interleukin-6, rheumatoid factor determination, anti-cyclic citrullinated peptide antibody determination, glycated hemoglobin determination, urine routine, fecal routine, etc.

Hypertensive patients monitor and control blood pressure before surgery, and diabetic patients monitor and control blood glucose before surgery.

Anesthesia department outpatient assessment: After completing preoperative examinations, patients assess surgical risks at the anesthesia department outpatient clinic and exclude surgical contraindications.

If preoperative examination results are abnormal, related departments will be consulted for guidance and treatment after admission. Patients with no obvious abnormalities in preoperative examination results and no surgical contraindications will enter the next process.

#### 3.2.2 Preoperative patient preparation

Before admission, the attending physician will explain the surgical process and possible complications in detail, inform the postoperative rehabilitation process, and patients will sign relevant informed consent forms after full understanding. The attending physician will notify patients at least one day in advance to fast from solid food 8 h before surgery, prohibit drinking 2 h before surgery, and arrive at the ward to handle admission procedures before 08:00 on the day of surgery. Complete drug allergy tests (antibiotics), prepare the surgical area, prevent foot infections of the affected limb (brush the foot with iodine twice), prepare items (bring drugs and imaging data for surgery), and confirm the surgical marking.

### 3.3 Official admission process

The attending physician will inquire about the detailed medical history, review the examination results, and issue preoperative medical orders after the patient reports.

#### 3.3.1 Preoperative

Empirical intravenous infusion of antibiotics is given 0.5 h before surgery or during anesthesia induction to prevent infection, and the commonly used antibiotics are first-generation cephalosporins or second-generation cephalosporins.

#### 3.3.2 Postoperative rehabilitation and discharge

Postoperative patients are instructed on home treatment and exercises.

Discharge criteria:

1. Good mental and dietary condition;
2. No sleep disorders;
3. Unobstructed bowel and bladder movements;
4. No nausea or vomiting;
5. No severe pain, with a resting VAS score of ≤3;
6. No redness or swelling of the wound, no bleeding or exudation;
7. Patients master the functional exercise methods, and the range of motion of the knee joint meets the functional requirements, with flexion greater than 90° and extension close to 0°;
8. Hb ≥ 90 g/L, WBC ≤ 4 × 10⁹/L, CRP ≤ twice the normal value;
9. Able to walk with the aid of a walker, taking no less than 15 steps per minute, and no less than 5 m.

#### 3.3.3 Post-discharge management

1. Change the dressing at a regular medical institution every 2–3 days until 14 days after surgery, observe the wound recovery, and prevent surgical incision infection and artificial joint infection;
2. Strengthen exercises, and caregivers should urge patients to perform functional exercises to prevent postoperative joint stiffness and poor joint function;
3. Abstain from alcohol, wear anti-slip flat shoes, avoid dangerous movements and actions to prevent periprosthetic fractures and joint dislocations;
4. Patients with concurrent internal diseases should follow medical advice to regularly visit relevant departments and regularly monitor blood pressure and blood glucose;
5. Persist in wearing compression stockings and icing the surgical area while awake for 1 month after surgery;
6. Regularly review knee joint x-rays at 1, 2, 3, 6, and 12 months after surgery to understand the prosthesis condition.

## 4 Perioperative management of day surgery for total knee arthroplasty

### 4.1 Preoperative management

#### 4.1.1 Preoperative education

Before surgery, patients will receive comprehensive perioperative plan information and be equipped with dedicated personnel to answer questions, aiming to reduce patients' uncertainty, tension, anxiety, and fear. If patients cannot independently overcome these negative psychological states before surgery, the medical team will provide necessary psychological support and guidance. For patients with excessive anxiety, we suggest taking sleep aid medication the night before surgery to ensure adequate sleep and rest. In addition, before surgery, patients will also receive educational advice to encourage them to stop smoking, drinking, and engage in appropriate physical activities.

Patients are instructed to perform adaptive training to facilitate early postoperative rehabilitation. The content includes: breathing exercises, coughing and expectoration training, bed defecation and urination training, bed turning training, ankle pump exercises, isometric contraction exercises of quadriceps, and straight leg raising exercises.

#### 4.1.2 Preoperative analgesic management

Patients take non-steroidal anti-inflammatory drugs such as celecoxib and acetaminophen orally 2 h before surgery for preemptive analgesia.

#### 4.1.3 Preoperative sleep management

Oral benzodiazepines can be given to help with sleep before surgery.

### 4.2 Intraoperative management

#### 4.2.1 Intraoperative analgesic management

Peripheral nerve block anesthesia is chosen for anesthesia. The anesthesiologist prepares a “cocktail,” the formula of which is: ketorolac tromethamine + ropivacaine + tranexamic acid + dexamethasone+ epinephrine + dexmedetomidine. The surgeon injects the “cocktail” into the local infiltration.

#### 4.2.2 Intraoperative blood management

Intra-articular injection and intravenous infusion of tranexamic acid; maintaining blood pressure at a relatively stable lower level; using a tourniquet during surgery.

#### 4.2.3 Surgical management

Minimally invasive surgical methods are adopted as much as possible; tissue damage is minimized during surgery; surgery time is shortened.

### 4.3 Postoperative management

#### 4.3.1 Postoperative analgesic management

Postoperative patients take acetaminophen (aniline class drugs) at a fixed dose of 1 g every 8 h, pregabalin (a γ-aminobutyric acid-like substance) 75 mg twice daily, and non-steroidal anti-inflammatory drugs (etoricoxib) 60 mg once daily. Dose adjustments were protocol-driven: acetaminophen was reduced to 650 mg/dose for patients with hepatic impairment (ALT >2 × ULN), and pregabalin was discontinued if sedation scores exceeded predefined thresholds. Local ice packs are applied to the surgical area to reduce swelling and pain, and flurbiprofen patches (40 mg/patches, replaced every 24 h) are applied near the surgical area. Standardized dosing was maintained for 97.3% of participants, minimizing confounding potential.

#### 4.3.2 Management of postoperative nausea and vomiting

Anticholinergic drugs: metoclopramide; 5-HT3 receptor antagonists: granisetron, ondansetron.

#### 4.3.3 Prevention of venous thromboembolism

Elevate the affected limb after surgery to prevent deep vein return obstruction;
Use intermittent pneumatic compression devices and gradient pressure stockings;
Subcutaneous injection of low molecular weight heparin (such as enoxaparin).

#### 4.3.4 Functional exercises

Ankle pump, quadriceps exercises, knee extension exercises, and bed-assisted knee flexion exercises.

### 4.4 Post-discharge management

#### 4.4.1 Post-discharge rehabilitation and follow-up

Monitor patients' rehabilitation exercises through telephone and online communication.

#### 4.4.2 Postoperative education

Emphasize possible postoperative situations and emergency response measures.

## 5 Surgical process

After anesthesia, apply a tourniquet, perform routine disinfection, and lay the drape. The surgical approach is taken with the affected knee joint in a flexed position, choosing the anterior midline approach of the knee joint, and the standard medial parapatellar approach. Incise the joint capsule along the inner edge of the quadriceps femoris, the inner edge of the patella, and the inner side of the patellar ligament. Incise the anterior medial joint capsule under the periosteum and remove the osteophytes on the medial edge of the tibial plateau. Extend the knee, flip the patella outward, release the lateral wrinkle of the patella and femur, explore and remove the proliferative osteophytes in front of the femoral condyles and around the patella, and remove the inflammatory proliferative synovial tissue. Flex the knee, resect the anterior cruciate ligament, remove the osteophytes at the distal end of the femoral condyles, and use a cobra hook to fully protect the skin, tendons, and patella on both sides. Then complete the osteotomy and prosthesis installation operations, count the gauze and instruments, and locally infiltrate the “cocktail” (the formula is the same as “4.2.1 Intraoperative analgesic management”) in the infrapatellar fat pad, the attachment points of the medial and lateral collateral ligaments, and the subcutaneous fat. For patients with posterior cruciate ligament substitution type prostheses, a drainage tube is left in place, while for those with posterior cruciate ligament retention type prostheses, no drainage tube is left. Fill with hemostatic gauze, suture the joint cavity, deep fascia, subcutaneous tissue, and perform continuous subcutaneous suturing of the incision, apply pressure dressing to the affected limb, release the tourniquet, and the surgery is completed. After the surgery, patients are returned to the ward.

## 6 Observation indicators

This study analyzes the relationship between many preoperative and intraoperative indicators and delayed discharge after day surgery for total knee arthroplasty. These indicators are divided into 6 modules: sociodemographic indicators, knee osteoarthritis-related indicators, preoperative auxiliary examination-related indicators, and surgery-related indicators.

Sociodemographic indicators: gender, age, body mass index (BMI), smoking history, hypertension history, diabetes history, stroke history, coronary heart disease history, osteoporosis history.

Knee osteoarthritis-related indicators: disease duration, knee flexion range of motion, knee flexion contracture degree.

Preoperative auxiliary examination-related indicators: pulmonary CT, pulmonary hypertension, electrocardiogram or potassium test, carotid artery color Doppler ultrasound, bilateral lower limb artery and vein color Doppler ultrasound, left ventricular systolic and diastolic function, cardiac ultrasound, hemoglobin, platelets, albumin, estimated glomerular filtration rate, glycated hemoglobin. Since the clinical auxiliary examination report results are numerous, this study summarizes and simplifies the report results based on experience to meet the needs of statistical analysis.

Surgery-related indicators: anesthesia grading, anesthesia method, surgery time, blood loss.

## 7 Sample size assessment

After multivariate logistic regression analysis, 5 indicators were finally included in the regression model. According to the 10 EPV principle (more than 10 events per variable) (27), the minimum effective sample size: *n* = 5 × 10 = 50 cases. In this study, there were 151 normal discharges and 82 delayed discharges. The smaller value is the 82 cases of the delayed discharge group, so the effective sample size is 82 cases, which is more than 50 cases and meets the sample size requirement.

## 8 Data collection

Cases meeting the study's inclusion and exclusion criteria were retrieved via the “Discharged Surgical Patients” module of the electronic medical record (EMR) system at Guizhou Provincial People's Hospital. The data collection process included the following steps:

### 8.1 Case identification

A structured query was performed using predefined keywords (e.g., diagnosis codes, surgical procedure codes) to screen all discharged surgical patients between [start date] and [end date].

### 8.2 Data extraction

Two trained researchers independently extracted variables (demographics, clinical parameters, surgical outcomes) using a standardized case report form (Supplementary Appendix 3). Discrepancies were resolved by consensus or adjudication by a third senior investigator.

### 8.3 Data cleaning

Missing or ambiguous entries were cross-verified with original medical records. Illogical values (e.g., implausible laboratory results) were flagged and re-evaluated by the clinical team.

### 8.4 Quality control

Inter-rater Reliability: A random 10% sample of cases underwent dual extraction to ensure consistency (*κ* > 0.85).

This protocol ensured adherence to STROBE guidelines for observational studies.

## 9 Statistical methods

Use WPS 2019 Excel software to establish the original database, and enter data through the electronic medical record system and review of clinical data. Import the completed Excel database into SPSS 26.0.0.0 software for statistical analysis. Measurement data are expressed in the form of mean ± standard deviation (Mean ± SD), and categorical data are expressed in the form of number and percentage [*n* (%)].

Division of continuous variables: Since there is no fixed standard on how to divide continuous variables in clinical research of logistic regression type (28), in this study, continuous variables are divided appropriately by observing the trend of data between groups, combined with clinical professional and statistical considerations, and measurement data composed of continuous variables and classification data after division are analyzed respectively.

Univariate analysis: For measurement data that meet the normal distribution and homogeneity of variance, perform group comparison *t*-test; if they do not meet the normal distribution, perform group comparison *t*-test on the data after data transformation that meets the normal distribution and homogeneity of variance, and perform Wilcoxon rank-sum test on the original data if they still do not meet the normal distribution after data transformation. For bidirectional unordered categorical data, perform chi-square test, and if the expected value of the cell is less than 5 in more than 1/5 of the total number of cells or the expected value of any cell is less than 1, perform Fisher's exact probability method. For unidirectional ordered categorical data, perform Mann–Whitney *U* test.

Multivariate analysis: Since the dependent variable is a categorical variable in logistic regression, which is more commonly used and easier to interpret the results (29), the indicators with *P* < 0.05 in the above univariate analysis (preferably categorical variables) are included in the multivariate logistic regression analysis. According to the relevant statistical research suggestions, the forward-LR method in stepwise regression is used for analysis. In the above statistical analysis, the significance level *α* is 0.05. According to the model obtained from the multivariate logistic regression analysis, a prediction formula is established, and the prediction probability and prediction grouping of each case are obtained. The prediction accuracy is evaluated using specificity, sensitivity, positive predictive value, and negative predictive value; the ROC curve is used to evaluate the multivariate logistic regression model, and the area under the curve (AUC) is closer to 1, indicating a better prediction classification effect of the model.

## 10 Results

### 10.1 Analysis of risk factors for delayed discharge after day surgery for total knee arthroplasty

**Figure 1:**
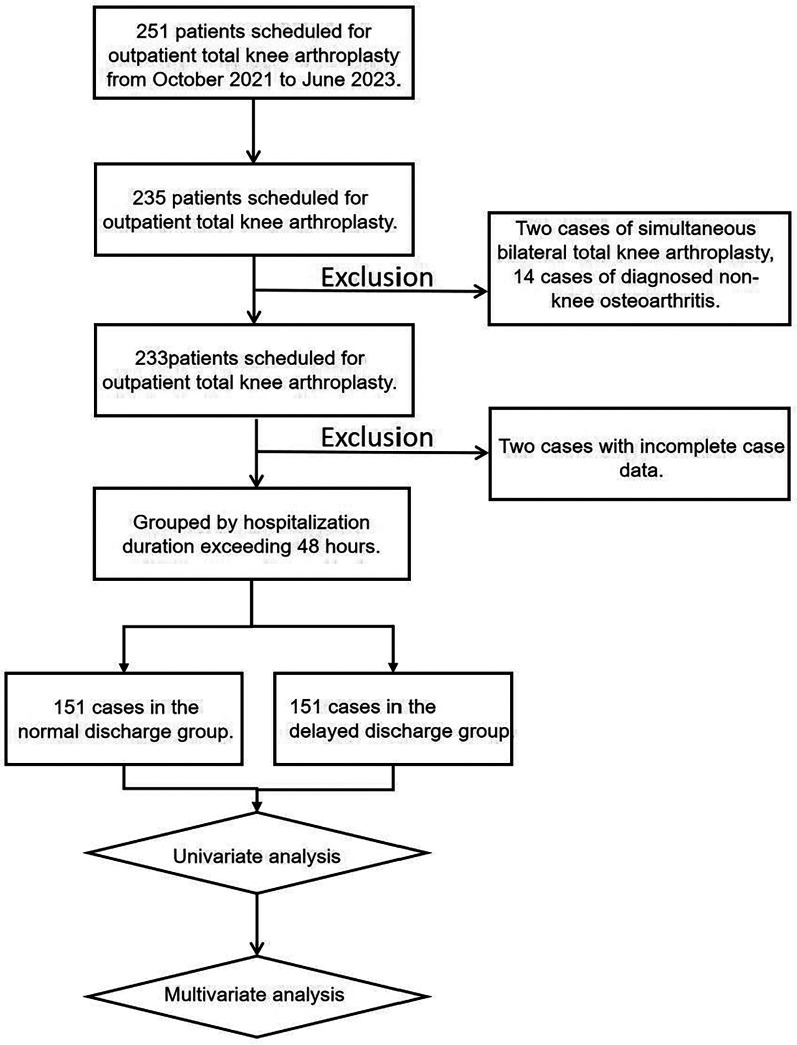
Flowchart of the study.

According to the inclusion and exclusion criteria, 18 patients who did not meet the criteria were excluded. A total of 233 patients scheduled for day surgery for total knee arthroplasty were included in this study, with 151 in the normal discharge group and 82 in the delayed discharge group. The specific flowchart is shown in Figure 1.

#### 10.1.1 Univariate analysis

Univariate analysis was conducted on sociodemographic indicators, knee osteoarthritis-related indicators, preoperative auxiliary examination-related indicators, and surgery process-related indicators.

#### 10.1.2 Univariate analysis of sociodemographic indicators between the normal discharge group and the delayed discharge group

The measurement data of age met the normal distribution and homogeneity of variance, and the group comparison *t*-test was performed. The results showed that the difference in age between the two groups was statistically significant (*P* = 0.042). The measurement data of BMI did not meet the normal distribution. After square root transformation, it met the normal distribution and homogeneity of variance. The group comparison t-test showed that there was no statistical difference in BMI between the two groups (*P* = 0.788). Chi-square tests were performed on bidirectional unordered categorical data, and the results showed that the difference in diabetes history (*P* = 0.046) between the two groups was statistically significant, while there was no statistical difference in gender (*P* = 0.378), smoking history (*P* = 0.215), hypertension history (*P* = 0.709), stroke history (*P* = 1.000), coronary heart disease history (*P* = 0.977), and osteoporosis history (*P* = 0.983) between the two groups. Mann–Whitney *U* tests were performed on unidirectional ordered categorical data, and the results showed that there was no statistical difference in age (*P* = 0.179) and BMI (*P* = 0.383) between the two groups. The results are shown in Table 1.

**Table 1:** Univariate analysis of sociodemographic indicators.

Indicator	All cases (*n* = 233)	Normal discharge group (*n* = 151)	Delayed discharge group (*n* = 82)	Z/X^2^/t	*P*
Gender, *n*				0.777	0.378
Male	38 (16.3%)	27 (17.9%)	11 (13.4%)		
Female	195 (83.7%)	124 (82.1%)	71 (86.6%)		
Age, years	63.17 ± 7.86	63.94 ± 7.84	61.76 ± 7.73	2.041	0.042*
Age, *n*				−1.345	0.179
<60 years	80 (34.3%)	49 (32.5%)	31 (37.8%)		
60–69 years	106 (45.5%)	67 (44.4%)	39 (47.6%)		
≥70 years	47 (20.2%)	35 (23.2%)	12 (14.6%)		
BMI, kg/m^2^	25.04 ± 3.87	25.08 ± 3.66	24.98 ± 4.25	0.269	0.788
BM, *n*				−0.872	0.383
<18.5 kg/m^2^	7 (3.0%)	4 (2.6%)	3 (3.7%)		
18.5 kg/m^2^ ≤ BMI < 24 kg/m^2^	83 (35.6%)	50 (33.1%)	33 (40.2%)		
24 kg/m^2^ ≤ BMI < 28 kg/m^2^	95 (40.8%)	66 (43.7%)	29 (35.4%)		
≥28 kg/m^2^	48 (20.6%)	31 (20.5%)	17 (20.7%)		
Smoking history, *n*				1.538	0.215
No	220 (94.4%)	140 (92.7%)	80 (97.6%)		
Yes	13 (5.6%)	11 (7.3%)	2 (2.4%)		
Hypertension history, *n*				0.139	0.709
No	144 (61.8%)	92 (60.9%)	52 (63.4%)		
Yes	89 (38.2%)	59 (39.1%)	30 (36.6%)		
Diabetes history, *n*				3.989	0.046*
No	211 (90.6%)	141 (93.4%)	70 (85.4%)		
Yes	22 (9.4%)	10 (6.6%)	12 (14.6%)		
Stroke history, n				<0.001	1.000
No	227 (97.4%)	147 (97.4%)	80 (97.6%)		
Yes	6 (2.6%)	4 (2.6%)	2 (2.4%)		
Coronary heart disease history, *n*				0.001	0.977
No	226 (97.0%)	147 (97.4%)	79 (96.3%)		
Yes	7 (3.0%)	4 (2.6%)	3 (3.7%)		
Osteoporosis history, *n*				<0.001	0.983
No	165 (70.8%)	107 (70.9%)	58 (70.7%)		
Yes	68 (29.2%)	44 (29.1%)	24 (29.3%)		

* Indicates *P* < 0.05.

#### 10.1.3 Univariate analysis of knee osteoarthritis-related indicators between the normal discharge group and the delayed discharge group

The measurement data of disease duration, knee flexion range of motion, and knee flexion contracture degree did not meet the normal distribution, and still did not meet the normal distribution after data transformation such as square root. The Wilcoxon rank-sum test was used, and the results showed that the differences in disease duration (*P* = 0.020) and knee flexion range of motion (*P* = 0.002) between the two groups were statistically significant, while the difference in knee flexion contracture degree (*P* = 0.077) was not statistically significant. Chi-square tests were performed on bidirectional unordered categorical data, and the results showed that the differences in disease duration (*P* = 0.012), knee flexion range of motion (*P* < 0.001), and knee flexion contracture degree (*P* = 0.006) between the two groups were statistically significant. The results are shown in Table 2.

**Table 2:** Univariate analysis of knee osteoarthritis-related indicators.

Indicator	All cases (*n* = 233)	Normal discharge group (*n* = 151)	Delayed discharge group (*n* = 82)	Z/X^2^/t	*P*
Disease duration, years	8.86 ± 6.59	8.21 ± 6.31	10.04 ± 6.98	−2.335	0.020*
Disease duration, *n*				6.338	0.012*
≤8 years	117 (50.2%)	85 (56.3%)	32 (39.0%)		
>8 years	116 (49.8%)	66 (43.7%)	50 (61.0%)		
Knee flexion range of motion,°	91.76 ± 8.63	92.85 ± 7.86	89.76 ± 9.62	−3.111	0.002**
Knee flexion range of motion, *n*				16.739	<0.001**
≥90°	171 (73.4%)	124 (82.1%)	47 (57.3%)		
<90°	62 (26.6%)	27 (17.9%)	35 (42.7%)		
Knee flexion contracture degree,°	8.69 ± 8.10	7.65 ± 5.50	10.61 ± 11.23	−1.771	0.077
Knee flexion contracture degree, *n*				7.562	0.006**
≤10°	185 (79.4%)	128 (84.8%)	57 (69.5%)		
>10°	48 (20.6%)	23 (15.2%)	25 (30.5%)		

* Indicates *P* < 0.05.

#### 10.1.4 Univariate analysis of preoperative auxiliary examination-related indicators between the normal discharge group and the delayed discharge group

The measurement data of hemoglobin, platelets, albumin, glomerular filtration rate, and glycated hemoglobin did not meet the normal distribution, and still did not meet the normal distribution after data transformation such as square root. The Wilcoxon rank-sum test was used, and the results showed that the difference in glomerular filtration rate (*P* = 0.011) between the two groups was statistically significant, while the differences in hemoglobin (*P* = 0.301), platelets (*P* = 0.072), albumin (*P* = 0.161), and glycated hemoglobin (*P* = 0.901) were not statistically significant. Chi-square tests were performed on bidirectional unordered categorical data, and the results showed that there was no statistical difference in pulmonary hypertension (*P* = 0.812), electrocardiogram or potassium test (*P* = 0.990), carotid artery color Doppler ultrasound (*P* = 0.326), and bilateral lower limb artery and vein color Doppler ultrasound (*P* = 0.715) between the two groups. In the bidirectional unordered categorical data of pulmonary CT, left ventricular systolic and diastolic function determination, and cardiac ultrasound, the number of cells with expected values less than 5 exceeded 1/5 of the total number of cells, so the Fisher's exact probability method was used. The results showed that there was no statistical difference in pulmonary CT (*P* = 0.091), left ventricular systolic and diastolic function determination (*P* = 0.708), and cardiac ultrasound (*P* = 1.000) between the two groups. The results are shown in Table 3.

**Table 3:** Univariate analysis of preoperative auxiliary examination-related indicators.

Indicator	All cases (*n* = 233)	Normal discharge group (*n* = 151)	Delayed discharge group (*n* = 82)	Z/X^2^/t	*P*
Pulmonary CT, *n*				4.912	0.091
Normal	221 (94.8%)	145 (96.0%)	76 (92.7%)		
Emphysema sign	9 (3.9%)	6 (4.0%)	3 (3.7%)		
Not done	3 (1.3%)	0 (0.0%)	3 (3.7%)		
Pulmonary hypertension, *n*				0.056	0.812
No	225 (96.6%)	145 (96.0%)	80 (97.6%)		
Yes	8 (3.4%)	6 (4.0%)	2 (2.4%)		
Electrocardiogram or potassium test, *n*				<0.001	0.990
Normal	223 (95.7%)	144 (95.4%)	79 (96.3%)		
Myocardial ischemia	10 (4.3%)	7 (4.6%)	3 (3.7%)		
Carotid artery color Doppler ultrasound, *n*				2.243	0.326
Normal	101 (43.3%)	66 (43.7%)	35 (42.7%)		
Arterial plaque	92 (39.5%)	63 (41.7%)	29 (35.4%)		
Not done	40 (17.2%)	22 (14.6%)	18 (22.0%)		
Bilateral lower limb artery and vein color Doppler ultrasound, *n*				0.672	0.715
Normal	176 (75.5%)	116 (76.8%)	60 (73.2%)		
Venous thrombosis, atherosclerotic plaques	31 (13.3%)	20 (13.2%)	11 (13.4%)		
Not done	26 (11.2%）	15 (9.9)	11 (13.4%)		
Left ventricular systolic and diastolic function, *n*				0.727	0.708
Normal	211 (90.6%)	136 (90.1%)	75 (91.5%)		
Left ventricular diastolic dysfunction	15 (6.4%)	11 (7.3%)	4 (4.9%)		
Not done	7 (3.0%)	4 (2.6%)	3 (3.7%)		
Cardiac ultrasound, *n*				0.380	1.000
Normal	224 (96.1%)	145 (96.0%)	79 (96.3%)		
Aortic stenosis	5 (2.1%)	3 (2.0%)	2 (2.4%)		
Not done	4 (1.7%)	3 (2.0%)	1 (1.2%)		
Hemoglobin, g/L	134.24 ± 14.92	134.83 ± 15.80	133.13 ± 13.16	−1.034	0.301
Platelets, 10⁹/L	236.48 ± 72.65	230.93 ± 74.80	246.70 ± 67.77	−1.796	0.072
Albumin, g/L	42.06 ± 3.86	42.40 ± 3.90	41.44 ± 3.73	−1.401	0.161
Glomerular filtration rate, ml/min	87.01 ± 15.97	85.05 ± 16.64	90.63 ± 14.05	−2.548	0.011*
Glycated hemoglobin, %	5.92 ± 0.68	5.89 ± 0.57	5.99 ± 0.86	−0.124	0.901

*Indicates *P* < 0.05.

#### 10.1.5 Univariate analysis of surgery-related indicators between the normal discharge group and the delayed discharge group

The measurement data of surgery time and blood loss did not meet the normal distribution, and still did not meet the normal distribution after data transformation such as square root. The Wilcoxon rank-sum test was used, and the results showed that the difference in surgery time (*P* < 0.001) between the two groups was statistically significant, while the difference in blood loss (*P* = 0.392) was not statistically significant. Chi-square tests were performed on bidirectional unordered categorical data, and the results showed that the difference in surgery time (*P* = 0.002) between the two groups was statistically significant, while the difference in anesthesia method (*P* = 0.604) was not statistically significant. Mann–Whitney *U* tests were performed on unidirectional ordered categorical data, and the results showed that there was no statistical difference in anesthesia grading (*P* = 0.742) between the two groups. The results are shown in Table 4.

**Table 4:** Univariate analysis of surgery-related indicators.

Indicator	Classification	All cases (*n* = 233)	Normal discharge group (*n* = 151)	Delayed discharge group (*n* = 82)	Z/X^2^/t	*P*
Anesthesia grading, *n*					−0.330	0.742
Grade 1		2 (0.9%)	2 (1.3%)	0 (0%)		
Grade 2		222 (95.3%)	143 (94.7%)	79 (96.3%)		
Grade 3		9 (3.9%)	6 (4.0%)	3 (3.7%)		
Anesthesia method, *n*					0.269	0.604
General anesthesia		216 (92.7%)	139 (92.1%)	77 (93.9%)		
Spinal anesthesia		17 (7.3%)	12 (7.9%)	5 (6.1%)		
Surgery time, min		82.64 ± 16.51	79.56 ± 15.72	88.29 ± 16.52	−4.060	<0.001**
Surgery time, *n*					10.055	0.002**
≤80 min		132 (56.7%)	97 (64.2%)	35 (42.7%)		
>80 min		101 (43.3%)	54 (35.8%)	47 (57.3%)		
Blood loss, ml		57.04 ± 23.07	55.5 ± 19.38	59.88 ± 28.57	−0.856	0.392

**indicates *P* < 0.01.

### 10.2 Multivariate analysis

#### 10.2.1 Multivariate analysis between the normal discharge group and the delayed discharge group

Since the dependent variable is a categorical variable in logistic regression, which is more commonly used and easier to interpret the results (29), the measurement data of age and glomerular filtration rate, and the categorical data of diabetes history, disease duration, knee flexion range of motion, knee flexion contracture degree, and surgery time with *P* < 0.05 in univariate analysis were included. The categorical data were assigned values, as shown in Table 5. The forward-LR method in stepwise regression was used for binary multivariate logistic regression analysis, and the results showed that disease duration (*P* = 0.017, OR = 2.055, 95% CI = 1.136–3.719), knee flexion range of motion (*P* < 0.001, OR = 3.164, 95% CI = 1.671–5.990), glomerular filtration rate (*P* = 0.010, OR = 1.028, 95% CI = 1.007–1.050), and surgery time (*P* = 0.020, OR = 2.002, 95% CI = 1.116–3.591) were independent risk factors, as shown in Table 6.

**Table 5:** Categorical data assignment.

Indicator	Assignment
Group	Normal discharge is 0, delayed discharge is 1
Diabetes	No is 0, yes is 1
Disease duration	≤8 years is 0, >8 years is 1
Knee flexion range of motion	≥90° is 0, <90° is 1
Knee flexion contracture degree	≤10° is 0, >10° is 1
Surgery time	≤80 min is 0, >80 min is 1

**Table 6:** Multivariate logistic regression analysis results of delayed discharge.

Item	B	Standard error	Wald	Significance	OR	95%CI
Disease duration	0.720	0.303	5.669	0.017*	2.055	1.136–3.719
Knee flexion range of motion	1.152	0.326	12.508	<0.001**	3.164	1.671–5.990
Glomerular filtration rate	0.028	0.011	6.716	0.010*	1.028	1.007–1.050
Surgery time	0.694	0.298	5.420	0.020*	2.002	1.116–3.591
Constant	−4.080	1.015	16.143	<0.01**	0.017	–

* Indicates *P* < 0.05, ** indicates *P* < 0.01.

#### 10.2.2 Risk prediction model for delayed discharge

According to the results of multivariate logistic regression analysis, the risk prediction model for delayed discharge is fitted: Logit (P) = −4.080 + 0.694 * surgery time + 0.720 * disease duration + 1.152 * knee flexion range of motion + 0.028 * glomerular filtration rate.

#### 10.2.3 Model evaluation

As shown in Table 7, the model predicted that the number of cases (A) in the normal discharge group was 134, and the actual number of cases (B) in the delayed discharge group was 47; the number of cases (D) predicted to be in the delayed discharge group was 35, and the actual number of cases (C) in the normal discharge group was 17. The sensitivity was D/(B + D) = 43%, indicating that only 43% of the patients who were actually delayed in discharge could be correctly predicted, which was less than 50%, and the sensitivity was low. The specificity was A/(A + C) = 89%, indicating that 89% of the patients who were actually normally discharged could be correctly predicted, which had good specificity. The positive predictive value was D/(C + D) = 67%, reflecting that the possibility of delayed discharge in patients predicted to be delayed in discharge was 67%, which was greater than 50%, and had a higher positive predictive value. The negative predictive value was A/(A + B) = 74%, reflecting that the possibility of normal discharge in patients predicted to be normally discharged was 74%, which was greater than 50%, and had a higher negative predictive value. Therefore, the model had higher specificity, positive predictive value, and negative predictive value, but the sensitivity was low.

**Table 7:** Actual and predicted grouping situation.

Actual grouping	Normal discharge group (*n* = 151)	Delayed discharge group (*n* = 82)
Predicted grouping		
Normal discharge group (*n* = 181)	134	47
Delayed discharge group (*n* = 52)	17	35

**Figure 2:**
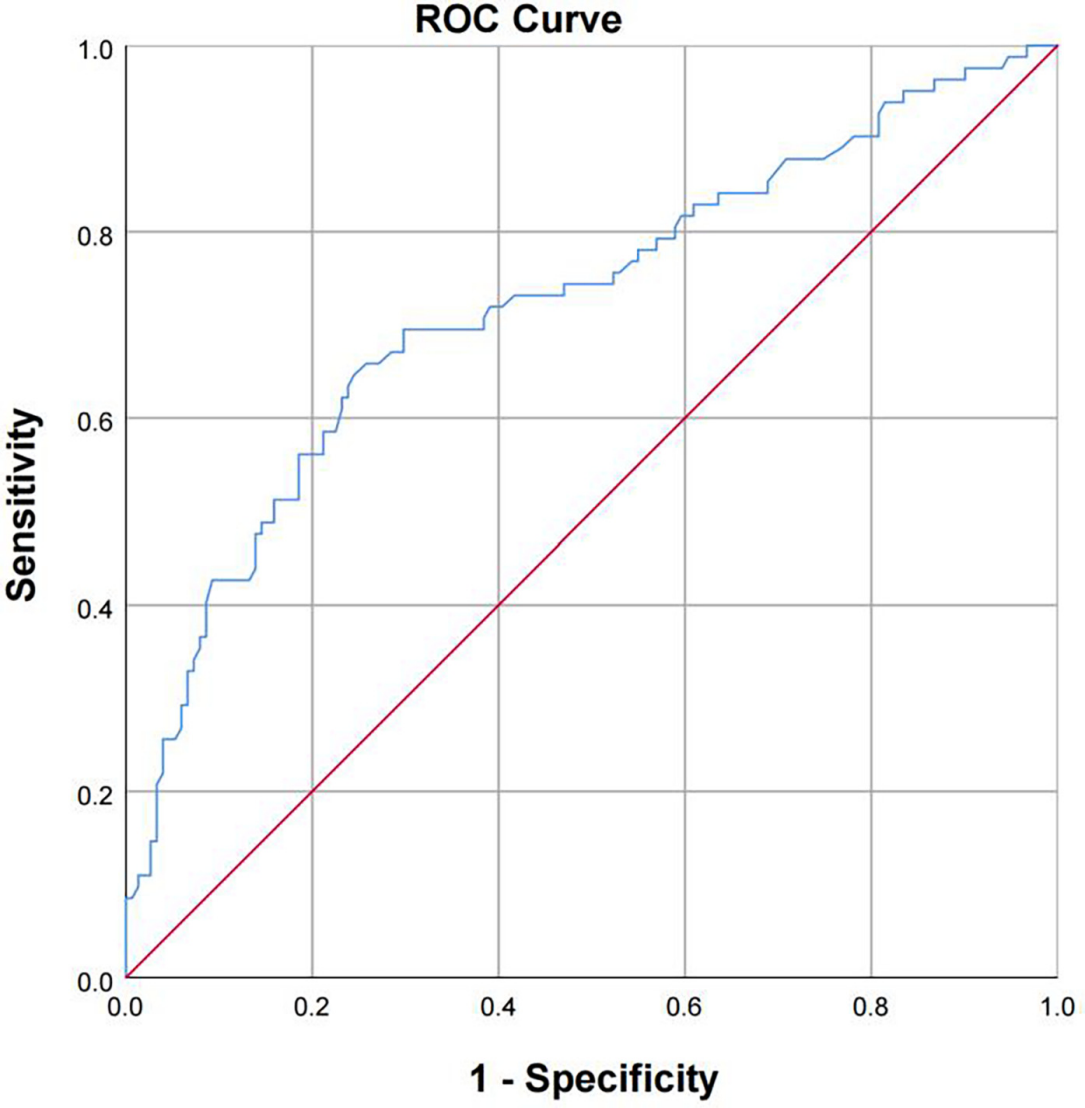
ROC curve.

The receiver operating characteristic curve (ROC) was used to evaluate the prediction classification effect of the model. The model was considered to have sufficient fit (*P* < 0.001). The area under the curve (AUC) was 0.722 (95% CI: 0.651–0.794), which was close to 1, indicating that the prediction classification effect of the model was good (Table 8; Figure 2).

**Table 8:** ROC curve evaluation.

AUC	Standard error	*P* (asymptotic)	95%CI (asymptotic)
0.722	0.036	<0.001**	0.651–0.794

** indicates *P* < 0.01.

## 11 Reasons for delayed discharge after day surgery for total knee arthroplasty

**Figure 3:**
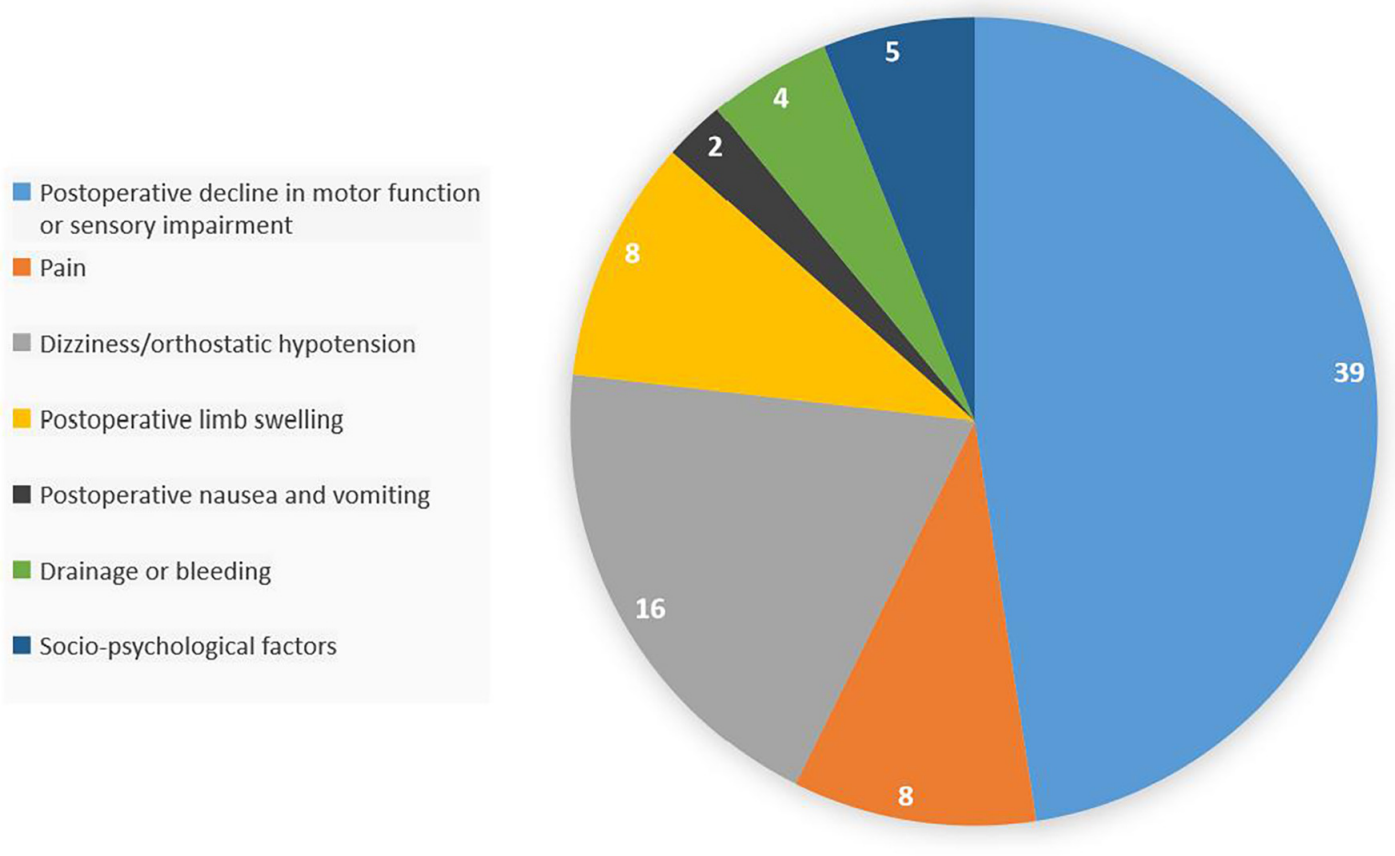
Reasons for delayed discharge after day surgery for total knee arthroplasty.

Through reviewing the medical records, postoperative medical orders, postoperative imaging and laboratory examinations, and telephone consultations, the reasons for delayed discharge after day surgery for total knee arthroplasty were understood. A total of 82 patients with delayed discharge, the main reason was postoperative motor function decline or sensory impairment (47.56%), followed by dizziness/orthostatic hypotension (19.51%), as shown in Table 9; Figure 3. For patients with postoperative motor function decline or sensory impairment, physical therapy and guidance were given, and patients were instructed to perform lower limb functional exercises (ankle pump, quadriceps exercises, knee extension exercises, bed-assisted knee flexion exercises), the specific content of functional exercises is shown in Supplementary Appendix 1. For patients with postoperative pain affecting timely discharge, medication was given according to postoperative analgesic management. For patients with dizziness or orthostatic hypotension, they were instructed to change body position slowly, or to use body position adaptation training, the specific content of the training is shown in Supplementary Appendix 2. For patients with postoperative limb swelling, the affected limb was elevated to promote venous blood return, and manual lymphatic drainage was performed if necessary. For patients with postoperative nausea and vomiting, antiemetic drugs were used for symptomatic treatment, and common drugs included benzamide class (metoclopramide). For patients with bleeding and exudation, sterile dressing change and wound observation were performed, and limb compression bandage was applied, and if necessary, the wound was debrided, the blood clot in the wound was cleared, and then sutured again. For patients affected by psychosocial factors, they were discharged after psychological counseling and agreed to delay discharge after surgery.

**Table 9:** Reasons for delayed discharge after day surgery for total knee arthroplasty.

Reason for delayed discharge after day surgery for total knee arthroplasty	Number of cases [*n*, (%)]
Postoperative motor function decline or sensory impairment	39 (47.56%)
Pain	8 (9.76%)
Dizziness/orthostatic hypotension	16 (19.51%)
Postoperative limb swelling	8 (9.76%)
Postoperative nausea and vomiting	2 (2.44%)
Bleeding and exudation	4 (4.88%)
Psychosocial factors	5 (6.10%)
Total	82 (100%)

## 12 Discussion

### 12.1 Risk factors for delayed discharge after day surgery for total knee arthroplasty

Based on literature research and the clinical characteristics of day surgery for total knee arthroplasty, this study divided cases into a normal discharge group and a delayed discharge group, and analyzed the risk factors for delayed discharge after day surgery for total knee arthroplasty from 40 indicators in four modules: sociodemographics, knee osteoarthritis, auxiliary examinations, and day surgery, respectively, using univariate analysis and binary multivariate logistic analysis. The results of univariate analysis showed that the measurement data of age (*P* = 0.042) and glomerular filtration rate (*P* = 0.011), and the categorical data of diabetes history (*P* = 0.046), disease duration (*P* = 0.012), knee flexion range of motion (*P* < 0.001), knee flexion contracture degree (*P* = 0.006), and surgery time (*P* = 0.002) had statistical differences between the two groups.

The relationship between delayed discharge and age. In the study of risk factors for delayed discharge after day surgery for total knee arthroplasty, Thomas et al. (18) conducted a retrospective case-control study including 2,313 patients from their own institution, and used binary logistic regression analysis to conclude that age of 65 years and above is an independent risk factor for patients transitioning from an outpatient status to an inpatient status and staying in the hospital for more than two nights. Turcotte et al. (30) also found in their retrospective case-control study that an increase in age would reduce the probability of discharge on the same day. The results of the univariate analysis of age in this study showed that the difference between the two groups was significant, and the average age of the normal discharge group was higher than that of the delayed discharge group, indicating that higher age is actually a protective factor against delayed discharge, which is contrary to our previous understanding. This discrepancy may stem from our cohort's younger mean age [63.17 ± 7.86 vs. 66.53 ± 7.01 in Thomas et al. (18)] and underrepresentation of high-risk elderly. This may be affected by the inclusion criteria of day surgery in this study. Thomas et al. (18) did not state the inclusion criteria for patient age in their study, and the upper limit of patient age was up to 93 years old. In a study on the anxiety status of elderly patients undergoing total knee arthroplasty, nearly 40% of patients felt severe anxiety when told to consider surgical treatment (31). In domestic regions, elderly people are less willing to accept joint replacement surgery due to various psychosocial factors, which makes the age distribution of patients included in this study concentrated in the lower age group (60–69 years old, 45.5%). Residual confounding by unmeasured variables (e.g., family support, preoperative mobility) correlated with age may also contribute. Moreover, in the subsequent multivariate analysis, the age factor was excluded due to the influence of other factors, indicating that the results of the univariate analysis of age are unstable, and future studies may need to include more elderly patients to reduce bias.

The relationship between delayed discharge and diabetes history. Diabetic patients undergoing surgical intervention will develop a state of insulin resistance and persistent hyperglycemia, increasing the risk of postoperative complications (32), and the hospital stay is relatively longer (33, 34). Shohat et al. (35) also found that postoperative hyperglycemia prolongs the hospital stay. Johnson et al. (36) conducted a retrospective study including 210,075 cases of day surgery for total knee arthroplasty and found that the risk factors for discharge after 24 h included a history of diabetes. In this study, diabetes history is a risk factor for delayed discharge, but the heterogeneity is not significant after adjustment for other factors. Similar to this study, several retrospective case-control studies on the relationship between hospital stay and internal diseases in patients undergoing total knee arthroplasty have also shown the same results (37, 38).

The relationship between delayed discharge and flexion contracture degree. Knee flexion contracture deformity is usually associated with late-stage knee arthritis (39–41), mainly due to the osteophytes formed on the posterior side of the distal femur affecting the extension of the knee joint (42), and the contracture of the iliotibial band and biceps femoris tendon further aggravates the flexion contracture of the knee joint (43). In a similar study, Matsumoto et al. (44) conducted a retrospective analysis of 158 patients who underwent unicompartmental knee arthroplasty, and the results of univariate analysis also showed that flexion contracture degree is a risk factor. A retrospective study showed that preoperative knee flexion contracture increased the probability of persistent pain after total knee arthroplasty (45), which may also affect the patient's higher pain in the short term after surgery, thereby delaying the discharge time.

In order to further analyze the independent risk factors for delayed discharge after day surgery for total knee arthroplasty, this study included the measurement data of age and glomerular filtration rate, and the categorical data of diabetes history, disease duration, knee flexion range of motion, knee flexion contracture degree, and surgery time with *P* < 0.05 in univariate analysis, and performed binary multivariate logistic regression analysis after assigning values to the categorical data. The results showed that disease duration, knee flexion range of motion, glomerular filtration rate, and surgery time were independent risk factors. In terms of auxiliary examinations, the higher the glomerular filtration rate, the higher the risk of delayed discharge after surgery (*P* = 0.010, OR = 1.028, 95% CI = 1.007–1.050). In terms of disease duration, patients with >8 years had a higher risk of delayed discharge than those with ≤8 years (*P* = 0.017, OR = 2.055, 95% CI = 1.136–3.719). In terms of knee flexion range of motion, patients with knee flexion range of motion <90° had a higher risk of delayed discharge than those with ≥90° (*P* < 0.001, OR = 3.164, 95% CI = 1.671–5.990). In terms of surgery time, patients with >80 min had a higher risk of delayed discharge than those with ≤80 min (*P* = 0.020, OR = 2.002, 95% CI = 1.116–3.591).

The relationship between delayed discharge and disease duration. In this study, patients with disease duration >8 years had a higher risk of delayed discharge than those with ≤8 years (*P* = 0.017, OR = 2.055, 95% CI = 1.136–3.719). Relevant studies (46, 47) have shown that disease duration is a risk factor for lower limb swelling and rehabilitation self-efficacy after TKA. Lower limb swelling often occurs after total knee arthroplasty, which can significantly prolong the patient's hospital stay. Swelling not only increases the burden of postoperative rehabilitation exercises for patients, but also may lead to increased pain and increased risk of infection. Therefore, we should pay full attention to postoperative lower limb swelling and take measures in time to shorten the patient's hospital stay as soon as possible.

The relationship between delayed discharge and knee flexion range of motion. A meta-analysis of the effects of preoperative rehabilitation on total knee arthroplasty showed that preoperative rehabilitation can effectively shorten the hospital stay (48). Calatayud et al. (49) found that preoperative high-intensity strength training can significantly improve the preoperative knee range of motion, thereby shortening the hospital stay and accelerating the physical and functional recovery after total knee arthroplasty. However, in these studies, the knee flexion range of motion was only described as an outcome indicator, and not analyzed as a preoperative factor. A recent study on the relationship between preoperative knee stiffness and postoperative clinical efficacy (50) showed that patients with smaller preoperative knee range of motion had better improvement in range of motion after total knee arthroplasty, but still had worse postoperative effects than patients with larger preoperative range of motion. This indicates that the smaller the preoperative knee flexion range of motion, the less favorable it is for the improvement of postoperative knee range of motion and functional recovery, leading to failure to meet the discharge standard of postoperative knee flexion degree greater than 90°, resulting in delayed discharge after day surgery for total knee arthroplasty.

The relationship between delayed discharge and glomerular filtration rate. Elderly patients often have renal cortical atrophy, reduction of renal units, decreased glomerular filtration rate, and decreased renal concentration function, which are manifestations of renal function decline. Renal function decline causes the excretion of anesthetic drugs to slow down (51), which may lead to postoperative nausea, vomiting, and other complications. The occurrence of these adverse reactions may prolong the patient's hospital stay and affect the recovery process. Therefore, in elderly patients, special attention should be paid to the dosage and pharmacodynamic monitoring of anesthetic drugs to avoid adverse consequences as much as possible.

The relationship between delayed discharge and surgery time. Papalia et al. (37) conducted a retrospective negative binomial regression analysis including 1,200 cases and concluded that surgery time is an independent risk factor for increased hospital stay after day surgery for total knee arthroplasty. Daniel et al. (36) and Gong et al. (52) also obtained the same results. In the above studies, surgery time was always analyzed as measurement data, but no stratification of time was performed. This study attempted to divide the surgery time into two groups based on the observed numerical range of surgery time between the two groups, with 80 min as the boundary. This is an empirical attempt based on mathematical characteristics. The result of this division is positive, so it can be better explained. The division method of this study can provide ideas for the division of surgery time in future related studies.

These risk factors may further impact long-term quality of life (QOL), as evidenced by international comparisons. A prospective European cohort study (53) found that higher BMI and lower education levels—both overlapping with our predictors of delayed discharge—were associated with poorer physical QOL at 3-month post-TKA. Notably, their stratified analysis revealed that smokers undergoing rehabilitation had worsening physical QOL trajectories, while depressive symptoms halted QOL improvement, suggesting that psychosocial interventions targeting these modifiable factors could synergistically optimize both discharge efficiency and postoperative wellbeing.

## 13 Limitations of the study

This study aims to analyze the risk factors for delayed discharge after day surgery for total knee arthroplasty, expanding the understanding of this surgical recovery model. However, the study has some limitations. First, as a retrospective case-control study, we cannot completely exclude the existing selection bias and information bias. Although we try to control known confounding factors as much as possible, there are still other unidentifiable or unmeasurable variables that may lead to delayed discharge after surgery. Secondly, the study sample comes from a single center, which may affect the universality of the results and may not represent all populations. Moreover, due to the limited number of patients undergoing day surgery, the statistical analysis may not be sufficient to detect all clinically relevant risk factors. Therefore, future studies should be designed as prospective multicenter studies and recruit more diverse patient groups, while comprehensively evaluating the impact of various biomedical factors on postoperative recovery.

## 14 Conclusion

This study identified four independent risk factors for delayed discharge following day surgery total knee arthroplasty: elevated glomerular filtration rate (OR = 1.028), disease duration >8 years (OR = 2.055), preoperative knee flexion <90° (OR = 3.164), and surgery time >80 min (OR = 2.002). These findings suggest that preoperative optimization targeting knee mobility improvement, standardized surgical workflows for time control, and exclusion of patients with prolonged disease courses could enhance day surgery efficiency. Surgeons should prioritize mitigating modifiable risks through technical refinement and patient selection guided by these predictors.

## Data Availability

The original contributions presented in the study are included in the article/Supplementary Material, further inquiries can be directed to the corresponding author.
