# Supplementary material for: Risk factors associated with the failure of day surgery for total knee arthroplasty a multivariate logistic regression analysis
Source: Front Surg. 2025 Jun 26;12:1597068. doi: 10.3389/fsurg.2025.1597068 (PMC12240994; doi:10.3389/fsurg.2025.1597068)

**Appendix**

**Appendix 1 Specific Content of Functional Exercises**

1.Ankle pump: By contracting the calf and calf muscles, rhythmically flex and extend the foot. Each time for 2-3 minutes, 2 to 3 times per hour. Repeat the exercise until recovery and the swelling of the calf and ankle has subsided.

2.Quadriceps exercises: While lying in bed, tighten the thigh muscles, try to straighten the knee, hold for 5 to 10 seconds, rest for 1 minute, and then repeat the above actions until fatigue.

3.Knee extension exercises: Place a folded towel under the heel, try to tighten the thigh muscles, and try to straighten the knee, hold the knee straight for 5 to 10 seconds. Repeat this action until fatigue.

4.Bed-assisted knee flexion exercises: Bend your knee, slide your foot towards the buttock direction, keep your heel on the bed. Try to bend your knee to the maximum range that can be tolerated for 5 to 10 seconds, and then straighten. Repeat until fatigue or the knee can be fully bent.

**Appendix 2 Specific Content of Position Adaptation Training**

Day 1: Raise the head of the bed to 30°, and instruct the patient to look straight ahead with both eyes, slightly retract the lower jaw, and maintain for more than 3 minutes.

Day 2: After getting used to it, the head of the bed can be raised to 60°-80°, observe whether there are any discomfort symptoms, and change to a supine position or give a soft pillow when resting.

Day 3: Adopt a progressive get up way, sit on the edge of the bed before standing; when standing, look straight ahead with both eyes, slightly retract the lower jaw, straighten the chest, keep the waist and back straight, stand with both legs, and keep the distance between the feet equal to the width of the shoulders, march in place, and then walk around the bed holding the bedrail.

**Appendix 3 Data Collection Form**


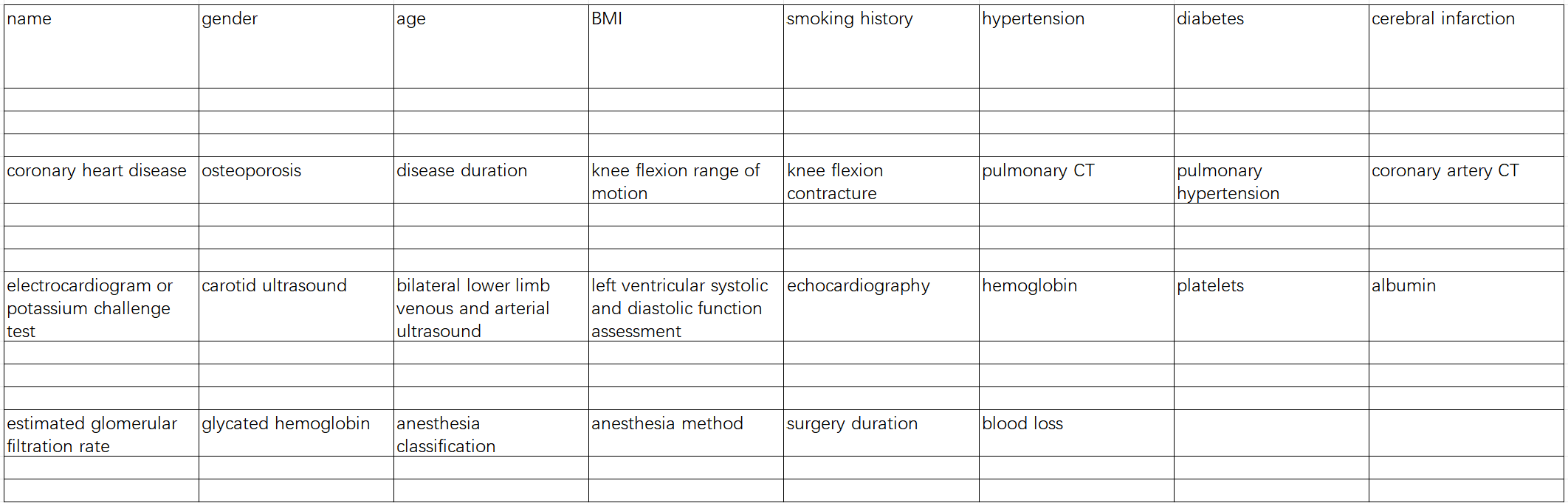

Supplement: Supplementary file 1 [file Table1.docx]
